# 62 Effect of Glycemic Variability on Infectious Outcomes in Patients with Severe Burn Injuries

**DOI:** 10.1093/jbcr/irad045.036

**Published:** 2023-05-15

**Authors:** David Hill, Kristine Hoang, Austin Ly, Xiangxia Liu, Sai Velamuri

**Affiliations:** Regional One Health, Memphis, Tennessee; Regional One Health, Memphis, Tennessee; University of Tennessee Health Science Center, Memphis, Tennessee; Firefighters Burn Center, Memphis, Tennessee; University of Tennessee, Memphis, Tennessee; Regional One Health, Memphis, Tennessee; Regional One Health, Memphis, Tennessee; University of Tennessee Health Science Center, Memphis, Tennessee; Firefighters Burn Center, Memphis, Tennessee; University of Tennessee, Memphis, Tennessee; Regional One Health, Memphis, Tennessee; Regional One Health, Memphis, Tennessee; University of Tennessee Health Science Center, Memphis, Tennessee; Firefighters Burn Center, Memphis, Tennessee; University of Tennessee, Memphis, Tennessee; Regional One Health, Memphis, Tennessee; Regional One Health, Memphis, Tennessee; University of Tennessee Health Science Center, Memphis, Tennessee; Firefighters Burn Center, Memphis, Tennessee; University of Tennessee, Memphis, Tennessee; Regional One Health, Memphis, Tennessee; Regional One Health, Memphis, Tennessee; University of Tennessee Health Science Center, Memphis, Tennessee; Firefighters Burn Center, Memphis, Tennessee; University of Tennessee, Memphis, Tennessee

## Abstract

**Introduction:**

Stress-induced hyperglycemia is a common manifestation of severe burn injury, due to increased catecholamines, glucagon, corticosteroids, and cytokines. The primary objective of this study was to evaluate the relationship between different measures of glycemic variability and infectious outcomes in burn patients.

**Methods:**

This retrospective electronic chart review included patients admitted to a single American Burn Association-verified burn

center for the year of 2020. Infectious complication was defined as occurrence of proven infection, autograft loss, or

death. Glycemic variability indices tested include standard deviation, mean amplitude of glycemic excursions, J-index,

and coefficient of variability. Demographics were compared utilizing Fisher’s exact or Mann-Whitney U test, depending

on dichotomy. Logistic regression analysis was utilized to control for revised Baux score. Likelihood ratio test statistic was

utilized to compare strength of association.

**Results:**

Three-hundred and ninety-two patients were admitted during 2020 and screened for inclusion. After applying exclusion

criteria, 112 patients remained in the study group. More than half were excluded for length of stay less than 3 days. Twenty-five

(22%) had at least one infectious complication (21 infections, 3 with graft loss, and 4 deaths). Revised Baux, age,

percent total body surface area burned, history of diabetes, and admission blood glucose were all higher in the group

with an infectious complication. Out of the indices tested, only mean glucose (Odds ratio, 95% confidence interval:

1.024, 1.004-1.045) and J-index (1.044, 1.002-1.087) were significantly associated with higher risk of an infectious

complication. Quantile analysis of mean glucose thresholds indicated > 150 mg/dL to have the strongest association with

infectious complications in burn patients.

**Conclusions:**

Although J-index was significantly associated with infectious complications, mean glucose was also and has a simpler

derivation.

**Applicability of Research to Practice:**

A liberal glucose control strategy should continue to be avoided in patients with burn injuries due to higher risk of infectious complication.

